# Valorization of the Residual Fraction of Coal Tailings: A Mineral Circularity Strategy for the Clay Ceramic Industry in the Carboniferous Region of Santa Catarina, Southern Brazil

**DOI:** 10.3390/ma17092131

**Published:** 2024-05-01

**Authors:** Wagner Benedet Rebelo, Alexandre Zaccaron, Emily Saviatto, Eduarda Fraga Olivo, Juliana Acordi, Fabiano Raupp-Pereira, Manuel Joaquim Ribeiro

**Affiliations:** 1 Post-Graduate Program on Materials Science and Engineering (PPGCEM), Universidade do Extremo Sul Catarinense, Avenida Universitária 1105, Universitário, Criciúma 88806-000, SC, Brazilalexandrezaccaron@hotmail.com (A.Z.); saviattoemily@gmail.com (E.S.); eduardaolivo@unesc.net (E.F.O.); julianaacordi@hotmail.com (J.A.); fraupp@unesc.net (F.R.-P.); 2 Mining Engineering Department, Universidade da Sociedade de Assistência aos Trabalhadores do Carvão SATC-UNISATC, R. Pascoal Meler, 73-Universitário, Criciúma 88805-380, SC, Brazil; 3 ProMetheus-Research Unit in Materials, Energy and Environment for Sustainability, Polytechnic Institute of Viana do Castelo, Rua Escola Industrial e Comercial de Nun’Álvares, 4900-347 Viana do Castelo, Portugal

**Keywords:** circular economy, clay ceramic, waste fraction, filter press cake, coal fines

## Abstract

Mineral extraction of coal in the carboniferous region of southern Santa Catarina (Brazil) plays a significant role in the regional economy. However, this activity has severe environmental impacts, with approximately 65% of the extracted mineral being discarded as a rejected material (deposited in landfills). The identification of the technological potential of these materials, based on the geological aspects of the extraction site and the beneficiation operations applied to obtain coal, provides the opportunity to add value to different residual fractions that can be reused. Thus, waste valorization, the main objective of this work, has recently become a strategy for the application of these minerals in the production of clay ceramics using a systematic approach named CPQvA, which means “classification, potentiality, quantity/viability, and applicability”. The use of these materials as secondary mineral sources can avoid the deposition of these materials in industrial landfills and help to reduce the pressure on landfills, which receive an average of four million tons of material annually. In this study, the residual fraction, part of the tailing from coal beneficiation, known as coal fines, was evaluated for formulation valorization in clay ceramics. This residual fraction was classified as non-hazardous (class II-A, non-inert). X-ray fluorescence spectrometry, X-ray diffraction, and thermogravimetric analysis were performed to characterize the moisture content, particle-size distribution, and coal content to be used in the development of different formulations using the residual fraction of coal tailings (ranging from 0 to 40%) that are of technological interest to the sector. Processing parameters, such as firing at different temperatures (750, 800, 850, and 900 °C), were also correlated with these formulations. The results were compared with those of a reference ceramic formulation from the local productive arrangement of Morro da Fumaça (Arranjo Produtivo Local Cerâmica Vermelha de Morro da Fumaça). The various relationships between the materials were characterized in terms of their thermal shrinkage, water absorption, and mechanical resistance. Leaching and solubilization environmental tests revealed that both the industrial standard formulation and formulations with the application of the residual fraction were classified as non-hazardous materials. Thus, the method of using a mining residual fraction in the formulation of clay ceramics proved to be beneficial for the circular economy in the regional mineral sector through productive and environmental gains; the primary mineral resource and energy consumptions and the impacts related to waste generation were reduced. The results of this study can be applied to similar situations in other parts of the world.

## 1. Introduction

The circular economy is an emerging concept aimed at extracting the maximum value from materials while minimizing waste and negative environmental impacts [1,2]. The mining industry plays a critical role in realizing the circular economy [3]. Academic research drives the sustainable development of mineral resources and the evolution of the circular economy [4]. Applying the fundamental concepts of the circular economy to mining waste presents a significant opportunity to reduce liability and increase the value of mining waste [5]. By adopting the principles of the circular economy, the mining industry can reduce waste and environmental impacts while increasing resource efficiency and value [6].

Industrial symbiosis is a collaborative approach among companies from different sectors to promote the valorization of waste, energy, and materials; the physical exchange of materials, energy, and waste creates economic, environmental, and social competitive advantages for all parties involved [7,8,9]. By establishing collaborative relationships and resource exchanges, different industries contribute to the advancement of the circular economy, reduce the need for natural resources (primary sources), minimize environmental impacts, and create economic and environmental benefits for all parties involved [10,11].

Coal mining in the carboniferous region of Santa Catarina, Brazil, plays an important role in the regional economy [12]. Brazilian coal reserves are distributed among the states of Paraná, Santa Catarina, and Rio Grande do Sul, with Santa Catarina accounting for 65% of the total run of mine (ROM) extracted [13]. Over the years, mining activities have had serious environmental impacts, leading to the degradation and abandonment of former mining areas [14,15]. Currently, approximately 2000 ha of land is occupied by coal waste dumps in the region, including active and inactive deposits, with an approximate volume of 58 billion cubic meters [16]. Inadequate mining practices, improper waste disposal, and a lack of regulation have resulted in severe environmental degradation and contamination [17,18].

Southern Santa Catarina is noteworthy because of its clay ceramic industry, mainly in the production of bricks and roof tiles, which has considerable socioeconomic importance for the region [19,20]. This sector gained prominence in the 1970s and 1980s owing to the abundance and quality of clay, which is the raw material base for ceramic production. The sudden growth had negative repercussions on the mining sector and the environment and exacerbated the region’s situation due to mineral extraction, which was performed at the time without minimum technical care and involved misinformation of industrialists [21].

The use of waste in manufacturing ceramic products is a promising approach for conserving natural clay resources [22]. Several studies indicate that the use of residual coal fractions is promising in clay ceramic applications [23,24,25,26,27,28,29]. The ceramic industry in the southern region of Santa Catarina has implemented circular economy principles, such as recycling and reusing discarded mineral resources, reducing energy consumption, and minimizing the generation of new waste [30].

The objective of this study is to assess the feasibility of applying fine coal waste from a mining company in the region to the production of clay ceramics using a well-known methodology referred to as “classification, potentiality, quantity/viability, and applicability” (CPQvA) [31]. This study contributes to the sustainable development of the ceramic industry and the entire southern region of Santa Catarina because information on the potential of the fine residual fraction of coal waste as a resource for circular economy initiatives was systematically obtained. In this sense, the main process considered in this study was the introduction of this fraction of coal residue, in different percentages, into the formulations of ceramic pastes typical of this region and, subsequently, the evaluation of the most appropriate incorporation content, thus facilitating a circular economy between two different industrial activities in the region.

## 2. Materials and Methods

**Figure 1:**
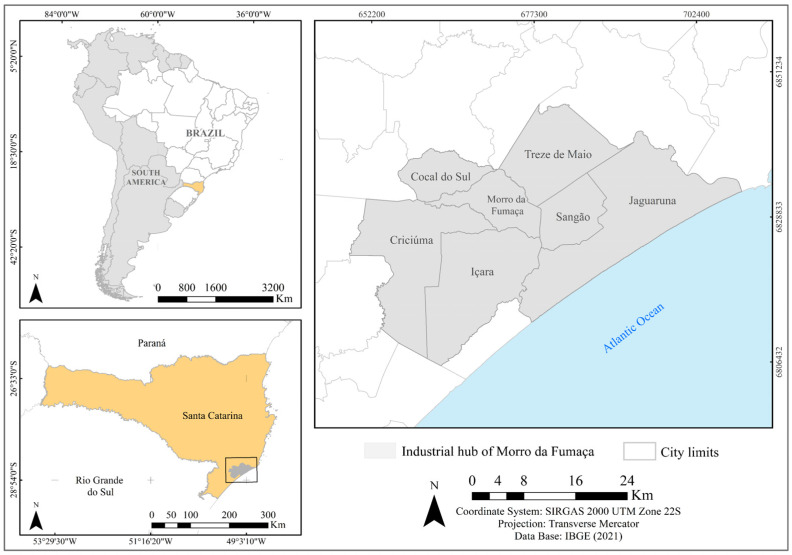
Geographic representation of the industrial hub of Morro da Fumaça, which is the focus in this study. Source: [35].

The industrial hub of Morro da Fumaça (Figure 1) is located in Santa Catarina, southern Brazil, and encompasses seven municipalities (Morro da Fumaça, Sangão, Içara, Jaguaruna, Treze de Maio, Criciúma, and Cocal do Sul), covering an area of 1,192,299 km^2^. It is included in the mesoregions of the Association of Municipalities of the Carboniferous Region (AMREC) and the Laguna Region (AMUREL). The mining–ceramic sector is crucial to the region, with clay being the primary raw material for the clay ceramic industry. Coal mining also has a significant impact on this region [32]; coal processing generates different types of residual fractions, and one of these fractions is clay [12], which can be a by-product of the production of clay ceramics [28,33,34].

The residual fraction was qualified using the CPQvA methodology proposed by Raupp-Pereira [31] to determine the prospects for the valorization of the material as a by-product in other applications. The following parameters for waste valorization were proposed:

I. Classification as hazardous or non-hazardous waste;
II. Potentiality and physical and chemical characteristics of the material;
III. Available quantity and feasibility of the waste;
IV. Applicability of the waste as a by-product in the ceramic industry.

Based on this innovative methodology, the development of all research activity involving these different valorization stages requires a well-defined time scope in terms of research evolution. The evaluation of the first three stages must be as quick as possible; however, the “applicability” parameter requires a strong investment of time and the availability of laboratory resources, which greatly conditioned this investigation work. Moreover, these four parameters were used to evaluate the feasibility of utilizing the residual fraction as a by-product in the production of clay ceramics for mineral circularity. This methodology has been widely applied in the scientific community [12,36,37,38,39,40,41,42].

The residual fine fraction from coal beneficiation, having homogeneous characteristics, was directly collected from the of a coal industry’s production plant; it is known as reject filter-press cake (BB:Fl(α)) in standard ceramic paste (STD). STD (approximately 40 kg) was obtained from an industry belonging to the productive hub of the “APL Clay Ceramic of Morro da Fumaça/SC”, and BB:Fl(α) (25 kg) was obtained from a coal company in the region following its processing in filter presses, which, concerning its beneficiation in flotation tanks, is the last stage of the fine and ultrafine reject treatment process.

Note that coal mining in the southern coal region of Santa Catarina is predominantly used for energy purposes and has considerable socioeconomic importance for this region [43,44]. Despite the global shift toward cleaner energy sources [45], shifting away from coal usage for energy generation will require a long time. In addition to the waste generated by the mining process, significant liabilities must be addressed. A promising approach is the exploration of the minerals in abandoned waste [46,47,48,49]. For example, the Brazilian Mineral Legislation, through Resolution ANM No. 85/2021 [50], stipulates that waste and tailings can be used if these are treated as ores. Another route is the industrial application of waste as a secondary mineral source, and in this case, the CPQvA methodology can be advantageous.

**Figure 2:**
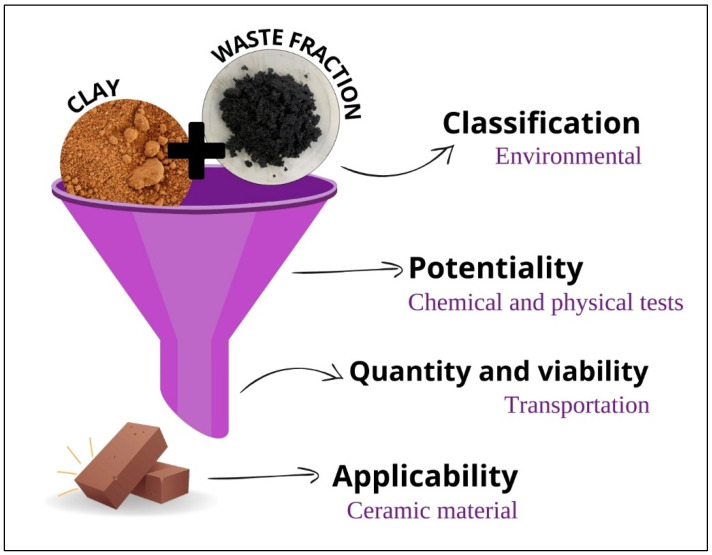
Representation of the CPQvA methodology for the valorization of the studied residual fraction, along with feasibility of application in a ceramic material.

Figure 2 schematically illustrates how the CPQvA methodology can assist in the valorization of the studied residual fraction within the clay ceramic industry.

### 2.1. Classification

The studied residual fraction was classified based on NBR 10004—solid waste classification [51]; NBR 10005—procedure for obtaining the leaching extract of solid wastes [52]; NBR 10006—procedure for obtaining the solubilized extraction of solid wastes [53]; NBR 10007—sampling of solid waste [54]; and the methodology based on SW 846-3050B [55].

### 2.2. Potentiality

To determine the potential of this residual fraction in clay ceramic pastes, both samples (BB:Fl(α) and STD) were initially analyzed to identify their chemical and mineralogical characteristics. For chemical composition evaluation, energy-dispersive X-ray fluorescence spectrometry was performed by using an EDX 7000 instrument (Shimadzu, Tokyo, Japan) as per the semi-quantitative method of oxides for powdered solid samples. Loss on ignition was performed at up to 950 °C as per the American Society for Testing and Materials D7348 standard (ASTM D7348) [56]. X-ray diffraction was performed for mineralogical characterization by using a LabX XRD 6100 instrument (Shimadzu, Tokyo, Japan) with Cu radiation, a tube voltage of 40 kV, a tube current of 30 mA, 2θ between 4° and 70°, a and speed of 0.02 °/s. Crystalline phases were identified using the PANalytical X’Pert HighScore Plus® software version 2.0 (Philips, The Netherlands). 

For differential thermal analysis and thermogravimetric analysis, a STA 449 F3 instrument (NETZSCH, Selb, Germany) was used, from 35 °C to 1100 °C, at a heating rate of 10 °C/min in a synthetic air atmosphere.

Particle size distribution was examined by performing laser diffraction using a CILAS laser granulometer instrument (reference 1064), working in the range of 0.04 to 500 µm for 60 s, and a dispersing agent based on sodium polyacrylate (Disperlan LP/G, Lamberti, Nova Odessa, SP, Brazil).

A combustibility analysis of the different samples was also conducted, where the higher heating value, based on the ASTM D 5865-13 standard [57], and volatile materials, based on ASTM D 3175 [58] and ASTM D 7582-15 [59], were determined.

For elemental analysis (CHNS/O), an elemental analyzer (CHN628 Series, LECO, St. Joseph, MI, USA) was used as per the ASTM D 5373 standard [60].

The sulfur forms, including sulfate and pyritic sulfur, were identified via instrumental analysis. The elemental analysis was conducted by performing combustion up to a temperature of 1350 °C using a CHN 628 SERIES SULFUR instrument (LECO, St. Joseph, MI, USA).

### 2.3. Quantity and Viability

**Figure 3:**
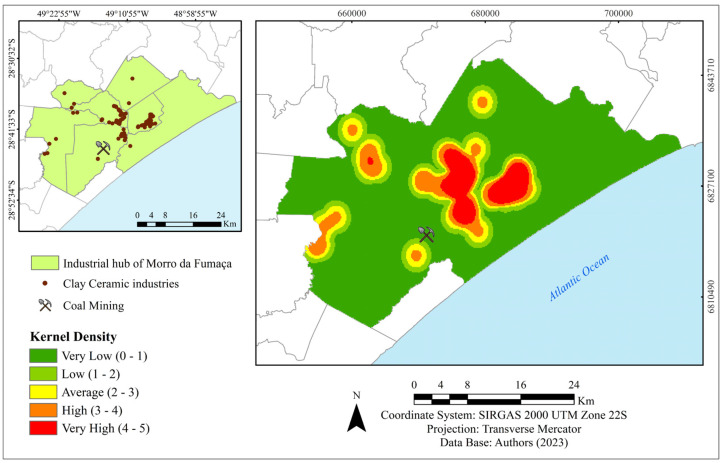
Map of the Morro da Fumaça Ceramic hub area (**left**) and schematic representation of industry density (**right**). Source: [61].

The study area comprised 120 ceramic industries that produce tiles, bricks, blocks, and other clay-based products (Figure 3). 

The carboniferous region is located in the southern part of the state, and within the ceramic hub, a mining company is located near the densest zone of the ceramic industry.

Although large coal deposits exist in coal-mining regions, the properties, mainly in the generated waste, are varied owing to the geology of the mining areas. The studied residual fraction was geologically inserted into the Rio Bonito Formation, where coal is mined from the Barro Branco layer [62]. Carbonaceous shale and diamictite are commonly found in carbonaceous matrices and arkose, siltstone, quartz sandstone, tonstein, and marl, which exist in fluviodeltaic, coastal, and platform marine environments [63]. All this geological variation, along with the beneficiation process, can result in rejected materials with distinct characteristics, even within the same outcrop.

The amount of waste (65% of the extracted ROM) generated because of coal concentration in Santa Catarina highlights that coal mining has environmental impacts and causes adverse effects on soil, water, and air [64]. Therefore, the feasibility of applying this material as a raw material or secondary source of mineral resources (by-products) favors circularity.

### 2.4. Applicability

To analyze the applicability of the material to clay ceramics, seven formulations were developed (Table 1) with different contents of BB:Fl(α) introduced into a clayey standard paste (STD) from a ceramic industry in the region.

To preserve the moisture content of each material and simulate the original conditions of the manufacturing process, formulations were developed on a wet basis. Therefore, before the seven formulations were prepared, the materials were subjected to a dehydration process to determine the moisture content of each sample; then, the total mass to be formulated was computed. The free moisture of the two materials (STD and BB:Fl(α)) was determined as per ASTM C324 [65] after the materials were dried in an oven (DeLeo n° 2211, 100 °C ±10 °C) for 24 h.

Once the free moisture value was computed, the masses required for the seven formulations were determined. The materials were weighed again, and appropriate aliquots of the reject were added to the ceramic paste for each pre-established formulation.

The test specimens were obtained using a vacuum-free extrusion process (Bonevau press 60 tons, plunger). Twenty-one cylindrical test specimens (Ø27 × 40 mm) were prepared for each formulation, and the standard dimension was evaluated with a length of 30 mm marked on a digital caliper (Digmess digital with precision of 0.01 mm). Mass values were obtained by using a scale (Marte AD 5002, with a maximum weight of 5000 g and precision of 0.01 g).

The shaped pieces underwent thermal treatment, starting with slow drying to avoid thermal shock and cracks, in an oven (DeLeo n° 2211, 60 °C ±10 °C) for 24 h. The drying shrinkage was determined as per ASTM C326-09 [66].

The test specimens were fired in a muffle furnace (laboratory scale with Novus n.1100 controller) at four temperatures (750, 800, 850, and 900 °C) for differentiated times with a thermal gradient of 5 °C/min and firing plateau time of 2 h. Subsequently, the pieces were measured again for firing shrinkage as per ASTM C326-09 [66] and subjected to water absorption tests based on technical standards 15270-2/2017 [67] and ASTM C20-00 [68].

The compressive mechanical strength was measured using a universal testing machine (EMIC DL 10000) at a speed of 1 mm/min.

Finally, the samples were evaluated for environmental quality to verify their corrosivity, reactivity, and leaching, as per ABNT, NBR 10004 [51], NBR 10005 [52], and NBR 10006 [53], based on standard methods for the examination of water and wastewater [69] and the United States Environmental Protection Agency [70] methodologies.

## 3. Results

### 3.1. Classification

The environmental classification of coal waste can depend on various factors, including its composition, potential for leaching toxic substances, and potential for environmental impact [71,72].

To classify the studied waste (residual fraction: BB:Fl(α)), the gross mass was initially evaluated via performed corrosivity tests (Table 2) and reactivity tests (Table 3). This residual fraction was classified as non-corrosive as it had a pH of 3.50 when mixed with water in a 1:1 weight ratio, thereby not exceeding the limit established in NBR 10004 [51]. The reactivity analysis showed that the fraction was non-reactive, as the sulfide content was 5.10 mg/kg, which is within the limit established in NBR 10004 [51]. Therefore, the fraction was classified as non-hazardous for these tests.

Subsequently, toxicity tests were performed by conducting leaching and solubilization tests. In the leaching test (Table 4), the parameters analyzed for the extract obtained did not show values above the maximum limits established in NBR 10005 [52]; thus, the fraction was characterized as non-toxic. In the solubilization test (Table 5), the concentrations of sulfates, total iron, manganese, and fluorides were higher than the maximum values established in NBR 10006 [53]; thus, the fraction was characterized as non-inert and non-hazardous (class II-A, non-inert).

### 3.2. Potentiality

The chemical characterization of the studied materials (Table 6) revealed the predominance of SiO_2_ + Al_2_O_3_ (between ~70% and ~80%), which may be associated with the presence of clay minerals [73]; these are oxides commonly found in clay ceramic pastes [21]. Alkali and alkaline earth oxides (K_2_O + CaO) are associated with the fusibility of the material [74], and their concentrations range from 2.72% to 5.24%, with high levels of the residual fraction. Chromophore oxide (Fe_2_O_3_ + TiO_2_) is associated with reddish coloration in ceramic pieces [75], and its concentration is 9.37–6.64%. The other oxide concentrations are <0.27%. SO_3_ is present in the residual fraction, and it is common in coal from the southern coal region of Santa Catarina, Brazil [76]. Finally, the loss on ignition reveals that the concentration of clay minerals, hydroxides, and organic matter in STD [77,78] is 9.45%, and that in BB:Fl(α), it is 18.63% owing to the high presence of organic matter [79,80]. The excess of these volatile fractions can cause the phenomenon known as “black core” [81,82] and increase the porosity in ceramic pieces [83,84] if the organic carbon is not completely decomposed.

**Figure 4:**
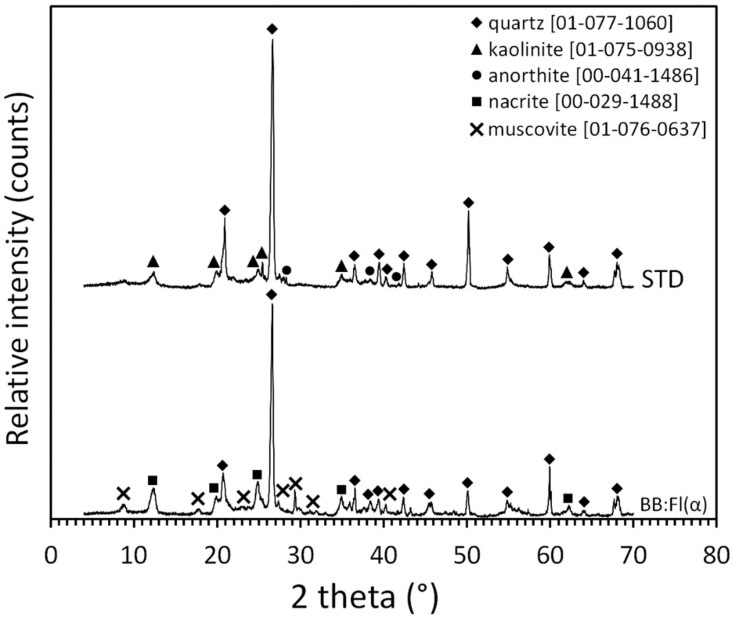
X-ray diffraction analysis of the studied samples for examining mineralogical composition.

The oxides of the chemical elements, in their most stable forms, can be observed in the X-ray diffraction patterns of the samples (Figure 4), where quartz (SiO_2_, reference code: 01-077-1060) can be observed in its free form in both samples. In STD, kaolinite ((Al,Mg,Fe)_4_(Si_4_O_10_)(OH)_8_; reference code: 01-075-0938), which is a clay mineral, is present. In BB:Fl(α), nacrite (Al_2_Si_2_O_5_(OH)_4_; reference code: 00-029-01488), which is a polymorphic clay mineral of kaolinite, was observed. In STD, anorthite (CaAl_2_Si_2_O_8_; reference code: 00-041-1486), which is a plagioclase feldspar, is also observed. In BB:Fl(α), muscovite (KAl_2_O_10_(OH, F)_2_; reference code: 01-076-0637), which is a clay mineral, is also observed.

**Figure 5:**
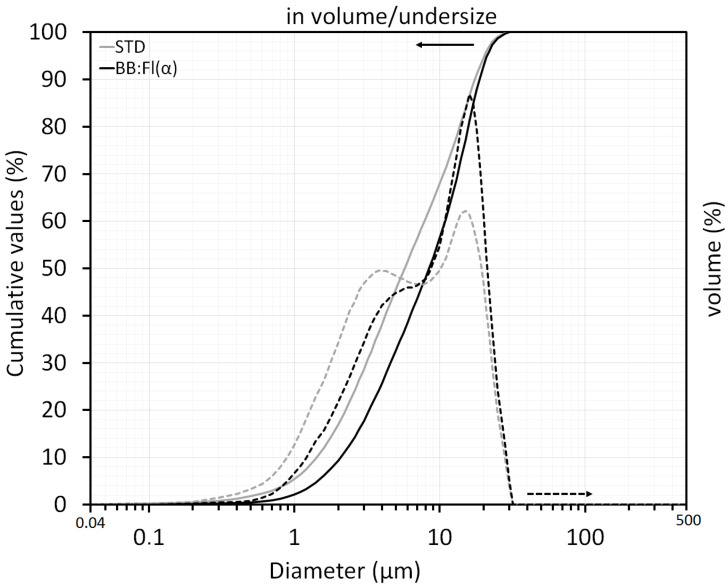
Cumulative distribution and particle size density of the studied samples.

The particle size distributions (Figure 5 and Table 7) of both samples were bimodal, with STD showing a smaller particle diameter compared with BB:Fl(α). Among the residues originating from the coal beneficiation process, the studied residue had a smaller diameter owing to the beneficiation method, which was flotation.

**Figure 6:**
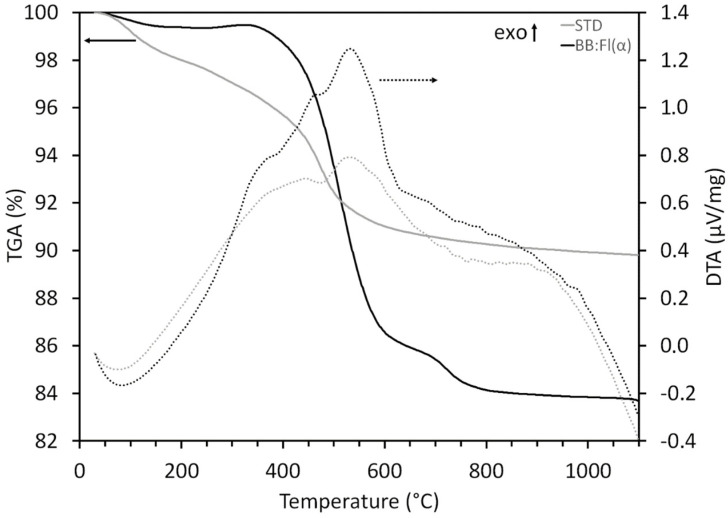
Differential thermal analysis (DTA) and thermogravimetric analysis (TGA) of the studied samples.

The thermal behavior of the studied samples (Figure 6) showed an initial mass loss (between 100 and 200 °C) in STD, attributed to the elimination of water present in the clays and the initiation of kaolinite dihydroxylation [85]. The thermal decomposition at 300–700 °C was mainly due to dehydroxylation (release of structural OH groups) of the clay minerals. A slight endothermic event occurred at 500 °C, which may be related to the amount of crystalline water with mass loss due to kaolinite dihydroxylation [86].

The thermal behavior of BB:Fl(α) exhibited an initial loss associated with superficial water, bound water, empty water, and gases [87]. The thermal decomposition at 300–800 °C occurred intensely and was possibly related to the dehydroxylation of the clay minerals present in the sample and the residual carbon, which gradually burned and released heat during the heating process [88].

The combustibility result (Table 8) indicated that the residual fraction of energy coal in BB:Fl(α) had a high calorific value (between 1858.0 and 5074.8 cal/g, as characterized in the southern coal region of Santa Catarina) [89]. These values can enhance the firing process in the ceramic sector [90].

Elemental analysis (Table 9) showed that carbon, hydrogen, and nitrogen were present in the organic matter. Carbon originated from the carbonates present in the organic matter, and hydrogen originated from the water content of clay minerals and the hydration of ferrous sulfate. The high percentage of ash (69.12%) was a result of the origin of the residual fraction analyzed in the beneficiation of coal concentrations from the ROM. Sulfur present in the residual fraction was in the form of sulfate (SO_4_), which is the most difficult type of sulfur to remove from the ROM by employing physical methods (density or washing) during the coal beneficiation processes because it is organically linked to the coal structure [91]. To examine the sulfur present in BB:Fl(α) (Table 10), note that sulfur in coal appears in three possible forms: pyritic (mineral), sulfate, and organic. Pyritic sulfur (0.95%), when in contact with O_2_ and H_2_O, forms ferrous sulfate (FeSO_4_) and sulfuric acid (H_2_SO_4_) and produces acid mine drainage, which is one of the main environmental problems of coal extraction [92]. Sulfate sulfur at a low concentration (0.13%) results from pyrite oxidation. Organic sulfur (0.17%) is chemically bound to coal molecules and has little effect on acid mine drainage [44].

### 3.3. Quantity and Viability

According to data acquired by Faraco (2022) [16], the coal mine located near the clay ceramic production sector generates 6000 tons/month of the clayey residual fraction. The Mineral Exploration Cooperative, which has 80 companies in the Morro da Fumaça Ceramic hub, handles an average of 35,000 tons/month of clay. Note that some ceramics have their own clay deposits, which lead to increased raw material consumption. Based on this, the viability of implementing mineral circularity with the valorization of this by-product in clay ceramic manufacturing was confirmed. 

It is estimated that the 120 industries in the Industrial hub of Morro da Fumaça have a production of 100,000,000 ceramic pieces/month, including tiles, bricks, blocks, and other clay-based products, with an average consumption of over 300,000 tons/month of clay. Based to the clayey residual fraction generated, there is the possibility of including it in ceramic pastes.

### 3.4. Applicability

#### 3.4.1. Technical Analysis

**Figure 7:**
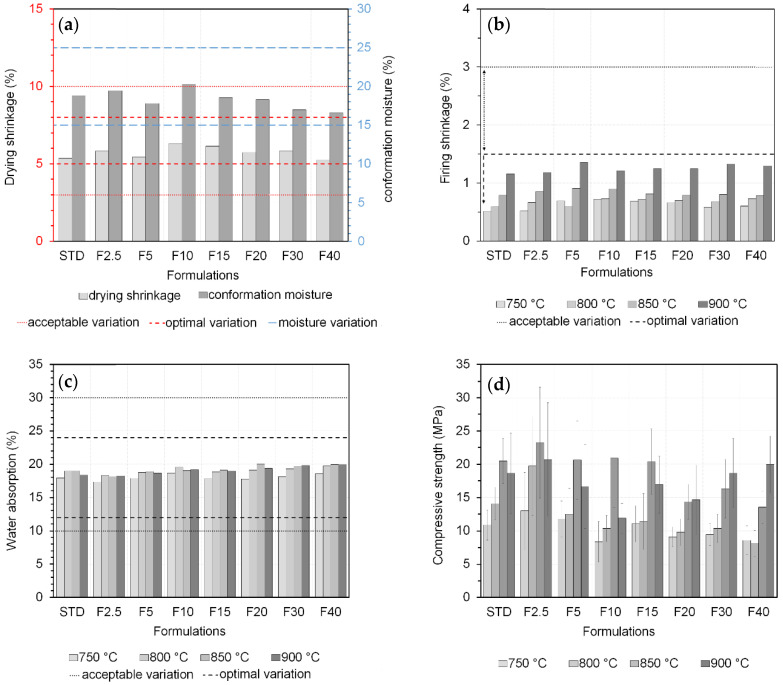
Technological characterization of the formulations: (**a**) moisture and drying shrinkage; (**b**) firing shrinkage; (**c**) water absorption; and (**d**) mechanical strength.

The technological characterization of the formulations displayed some of the properties of the developed mixtures. The moisture and drying shrinkage (Figure 7a) indicated pieces shaped within the limits proposed by Dondi (2003) [93] (between 15 and 25%). The optimal variation for the drying shrinkage was 5–8%, and all formulations met this criterion. The optimal value for the firing shrinkage was <1.5 (Figure 7b), and all formulations met this criterion. Water absorption (Figure 7c) was also within the optimal range of 12–24%. Finally, a mechanical strength test (Figure 7d) was conducted to compare the formulations; the mechanical strength increased as the sample became denser. Porosity is known to negatively influence strength, and increasing the temperature promotes piece densification, leading to increased compression resistance [94]. In this study, increasing the residual fraction content tended to decrease the mechanical strength. This property is strongly influenced by porosity, which reduces the number of effective surfaces where loads are applied. Note that almost all residues usually studied in ceramic manufacturing show a decreasing mechanical strength with an increasing content [95,96,97].

The presence of clay minerals in the residual fraction of coal tailings can be a determining factor in its interaction with the ceramic paste. It can be observed that for the firing shrinkage and water absorption tests, all formulations showed great similarity. In terms of water absorption, there was a slight tendency towards an increase as the waste was incorporated: <19% for STD and between 18.5 and 20% for F40. This is associated with the high loss on ignition (18.63%, Table 6), originating from the carbon content in the residual fraction of coal tailings.

The Industrial hub of Morro da Fumaça is a large manufacturer of ceramic products. According to studies conducted in this region, for hollow ceramic blocks for non-load-bearing masonry, they exhibit a mechanical resistance of 1.52 MPa [98] and 3.8 MPa [86] when fired at a temperature of 850 °C—commonly applied by ceramic industries. These values meet Brazilian legislation for this type of material [99].

#### 3.4.2. Environmental Analysis

Corrosivity and reactivity tests were conducted for the STD, F2.5, and F20 samples fired at 850 °C (the usual temperature for firing clay ceramics) for comparison. The purpose of this analysis, especially in the case of the samples containing the studied residual fraction, was to verify whether the materials are hazardous or have the ability to transfer substances to the extracts.

The corrosivity (Table 11) and reactivity (Table 12) results indicated that all samples were non-corrosive and non-reactive.

The leaching test (Table 13) was performed to assess the material’s ability to transfer the elements or organic and inorganic substances present inside the pieces to the extract outside through dissolution. In the leached extract from STD with the other formulations (F2.5 and F20), concentrations above the maximum permitted limit set forth in Annex F were not detected. Therefore, as the test specimens did not exhibit hazards (corrosivity, reactivity, and toxicity), they were classified as non-hazardous.

The aim of the solubilization tests (Table 14) was to compare the material’s ability to dissolve in water with that in the presence of the residual fraction. In the STD sample, aluminum, iron, and manganese concentrations were above the maximum allowable limits, indicating that this material has the ability to dissolve in water. In F2.5, only the manganese concentration was above the maximum value, indicating that aluminum and iron were inhibited by the addition of the residual fraction. In contrast, F20 still had manganese concentrations exceeding the limits; however, other elements also appeared to exceed the limits, such as fluoride, selenium, and sulfates. Therefore, the formulations were classified as non-inert in all cases.

Phytotoxicity tests conducted using *Allium cepa* L. as a bioindicator at different concentrations in the same carboniferous region revealed that 16% of the residual fraction of coal tailings in clay ceramics did not show toxicity [100].

## 4. Conclusions

To search for environmentally appropriate alternatives with economic potential for the disposal of the fine residual fraction from coal beneficiation tailings, the possibility of valorizing the fine residual fraction by applying it in clay ceramic paste was studied by using different formulations and firing at different temperatures.

The CPQvA methodology used systematically as a multicriteria parameter for the valorization of the residual fraction proved to be extremely effective.

The studied residual fraction was classified as non-hazardous (class II-A, non-inert), indicating that this material can be used for clay ceramic production.

The potential for valorization lies in the significant clayey residual fraction identified in the material from coal beneficiation. Although volatile materials were present, their use in controlled amounts did not negatively affect the manufacturing of ceramic products.

The quantity and viability proved to be beneficial as the amount of this material generated by the coal-mining company can meet the demands of the entire ceramic industry in the Morro da Fumaça region. An appreciable alternative is the creation of a central paste-production facility with the cooperative development of formulations using this residual fraction. 

The evaluated technological values were within acceptable parameters for clay ceramic manufacturing. The use of 2.5% of the residual fraction of coal tailings did not negatively alter the original properties of the clay ceramic paste, thereby inhibiting the concentrations of iron and aluminum and exhibiting high practical application viability. Finally, we can state as a result of this study, that this method of using coal tailings is potentially applicable in other regions or countries with similar, or even different, coal compositions.

## Figures and Tables

**Table 1:** Formulations developed for the study (STD: standard formulation; F: new formulations).

Materials	Formulations (%w)							
	STD	F2.5	F5	F10	F15	F20	F30	F40
STD	100	97.5	95	90	85	80	70	60
BB:Fl(α)	0	2.5	5	10	15	20	30	40

**Table 2:** Corrosivity analysis of the residual fraction.

Parameter	BB:Fl(α)	Recommended Value	L.Q.
pH in water (1:1)	3.50	2 to 12.4	0.10

L.Q. = limit of quantification.

**Table 3:** Reactivity analysis of the residual fraction.

Parameter	BB:Fl(α) (mg/kg)	Releasable Limit per kg of Waste	L.Q.
Sulfide	5.10	500 mg	40.0

L.Q. = limit of quantification.

**Table 4:** Leaching results of the residual fraction.

Parameter	BB:Fl(α) (mg/L)	Maximum Leached Limit (mg/L)	L.Q.
Leached pH	3.50	-	-
Lead	0.03	1.0	0.05
Barium	0.20	70.0	0.10
Cadmium	N.D.	0.5	0.01
Silver	N.D.	5.0	0.01
Arsenic	<0.001	1.0	0.01
Fluoride	0.65	150.0	0.10
Mercury	<0.001	0.1	0.001
Selenium	<0.001	1.0	0.001

L.Q. = limit of quantification; N.D. = not detected.

**Table 5:** Solubilization results of the residual fraction.

Parameter	BB:Fl(α) (mg/L)	Maximum Solubilized Limit (mg/L)	L.Q.
Solubilized pH	5.02	-	-
Sulfates	410.00	250.0	10.0
Chlorides	97.16	250.0	0.10
Phenol	N.D.	0.01	0.01
Total Iron	22.80	0.3	0.02
Manganese	0.79	0.1	0.01
Copper	0.01	2.0	0.01
Zinc	0.09	5.0	0.01
Aluminum	N.D.	0.2	0.10
Lead	N.D.	0.01	0.001
Sodium	30.94	200	0.01
Cadmium	N.D.	0.005	0.0001
Silver	N.D.	0.05	0.01
Barium	<0.01	0.7	0.10
Arsenic	<0.001	0.01	0.001
Fluoride	1.62	1.5	0.10
Mercury	<0.001	0.001	0.001
Nitrate Nitrogen	N.D.	10.0	0.10
Selenium	<0.001	0.01	0.001

L.Q. = limit of quantification; N.D. = not detected.

**Table 6:** Chemical composition, in terms of oxides, determined by performing X-ray fluorescence spectrometry on the studied samples.

Oxides (%)	SiO_2_	Al_2_O_3_	Fe_2_O_3_	K_2_O	TiO_2_	CaO	SO_3_	Others	LOI
STD	60.85	17.39	7.65	2.40	1.72	0.32	-	0.22	9.45
BB:Fl(α)	49.19	19.86	5.08	3.44	1.56	1.80	0.27	0.17	18.63

LOI: loss on ignition.

**Table 7:** Size distribution and average particle diameter of the studied sample.

Raw Material	Diameter (µm)			
	D10	D50	D90	Average
STD	1.43	5.73	17.48	7.85
BB:Fl(α)	2.08	8.44	18.75	9.59

**Table 8:** Characterization of the studied samples as fuel.

Parameters	Calorific Value (cal/g)	Volatile Matter (%)	Hygroscopic Moisture (%)
STD	117.00	7.01	2.69
BB:Fl(α)	983.00	10.56	1.36

**Table 9:** Elemental analysis (CHNS/O) of the residual fraction studied.

Parameters (%)	BB:Fl(α)
Carbon	21.84
Hydrogen	2.03
Nitrogen	0.70
Total Sulfur	1.25
Oxygen	5.06
Ash Content	69.12

**Table 10:** Determination of the sulfur content in the residual fraction studied.

Sulfur Content Determination (%)	BB:Fl(α)
Total	1.25
Pyritic	0.95
Sulfatic	0.13
Organic	0.17

**Table 11:** Results of the corrosivity analysis for the STD, F2.5, and F20 samples.

Parameter	Results (mg/L)			Recommended Value
	STD	F2.5	F20	
pH in water (1:1)	5.74	5.51	6.46	2.0 a 12.4

**Table 12:** Results of the reactivity test for the STD, F2.5, and F20 samples.

Parameter	Results (mg/kg)			Releasable Limit per kg of Waste
	STD	F2.5	F20	
Sulfide	0.18	0.28	1.2	500 mg

**Table 13:** Leaching results of the STD, F2.5, and F20 samples.

Parameter	Results (mg/L)			Maximum Leached Limit (mg/L)
	STD	F2.5	F20	
Arsenic	0.017	<0.010	<0.010	1.0
Barium	0.692	0.479	0.205	70.0
Cadmium	<0.010	<0.010	<0.010	0.5
Lead	<0.010	<0.010	<0.010	1.0
Total chromium	<0.010	<0.010	<0.010	5.0
Total fluoride	0.24	0.17	1.26	150.0
Silver	<0.010	<0.010	<0.010	0.1
Selenium	<0.010	0.012	0.03	5.0
Mercury	<0.0002	<0.0002	<0.0002	0.1

**Table 14:** Solubilization results of the STD, F2.5, and F20 samples.

Parameter	Results (mg/L)			Maximum Solubilized Limit (mg/L)
	STD	F2.5	F20	
Aluminum	1.249	0.134	0.115	0.200
Arsenic	<0.010	<0.010	<0.010	0.010
Barium	<0.700	0.062	0.027	0.700
Cadmium	<0.005	<0.005	<0.005	0.005
Lead	<0.010	<0.010	<0.010	0.010
Chlorides	<0.010	<0.010	0.95	250.0
Copper	<0.010	<0.010	<0.010	2.0
Total chrome	<0.010	<0.010	<0.010	0.050
Iron	0.311	0.106	0.019	0.300
Fluoride	0.12	0.33	1.56	1.50
Manganese	0.297	0.910	0.881	0.100
Nitrate	0.016	0.217	0.117	10.0
Silver	<0.010	<0.010	0.0108	0.050
Selenium	<0.010	<0.010	0.047	0.010
Sodium	3.414	6.100	8.399	200.0
Sulfates	48.88	113.68	611.6	250.0
Surfactants	0.113	0.084	0.158	0.5
Zinc	0.013	0.056	0.046	5.0
Mercury	<0.0002	<0.0002	<0.0002	0.001

## Data Availability

Data are contained within the article.
